# Multi-Order Mode Excitation and Separation of Ultrasonic Guided Waves in Rod Structures Using 2D-FFT

**DOI:** 10.3390/s23208483

**Published:** 2023-10-16

**Authors:** Gang Li, Jing Zhang, Juke Cheng, Kang Wang, Dong Yang, Ye Yuan

**Affiliations:** 1 Department of Civil Engineering, Hefei University of Technology, Hefei 230009, China; 2021170713@mail.hfut.edu.cn (G.L.);; 2 Earthquake Engineering Research & Test Center, Guangzhou University, Guangzhou 510006, China; yangd@gzhu.edu.cn; 3 Department of Civil Engineering, The University of Hong Kong, Pokfulam Road, Hong Kong, China

**Keywords:** ultrasonic guided wave, two-dimensional Fourier transform, multi-order modes, mode separation, frequency-wavenumber ridge extraction

## Abstract

The ultrasonic guided wave technique is extensively used for nondestructive structural testing, and one of the key steps is to extract a single mode with certain purity from multi-order mixed modes. In this paper, the propagation of ultrasonic guided waves in the cylindrical rod is simulated first; the appropriate broadband excitation signal is selected to excite the multi-order modes in a specific frequency range; and the time–space signal containing multi-order modes is converted to the frequency-wavenumber domain signal by two-dimensional Fourier transform. In the frequency-wavenumber domain, the frequency-wavenumber ridge is extracted from the multi-mode frequency-wavenumber domain based on the dynamic programming method, and then the time-domain signal corresponding to a single mode can be reconstructed. By comparing the excited multi-order mode and the separated single mode with the theoretical results, it is observed that the two results are consistent. Thus, the employed mode-excitation method can accurately excite the multi-order modes in rod structures. Furthermore, the proposed method enables the separation of a single-mode wave with high purity, providing a foundation for future utilization of isolated modes.

## 1. Introduction

Ultrasonic guided waves are widely used in the health monitoring of pipelines, plates, rods and other structures due to their long propagation distance, fast propagation speed and small attenuation [1,2,3,4,5]. When the ultrasonic wave propagates in the structure, the boundary will cause wave reflection and mode conversion [6]. Different structures and the existence of damage will lead to different guided wave modes [7]. In addition, different excitation locations and frequencies of excitation signals could excite guided wave modes of different frequency ranges [8]. Additionally, even at the same location and frequency, different modes exhibit variations in group velocity and phase velocity [9], leading to mode aliasing in time domain signals and posing challenges for the practical application of ultrasonic guided waves. Consequently, a prerequisite for utilizing ultrasonic guided waves in structural health monitoring is to investigate the propagation characteristics of guided waves in a specific structure [10], as well as to stimulate and extract the desired modes [11,12,13,14].

Since the frequency of the excitation signal cannot contain only a single frequency, the actual ultrasonic guided wave signal is often very complicated. This complexity shows the multi-modal characteristics of guided waves from the perspective of guided wave modes [15,16]. Guided wave dispersion curves can be extracted from time-transient measurements using time-frequency representations (TFRs). However, any TFR is limited by the time-frequency uncertainty principle [17]. To address this problem, De Marchi et al. [18] proposed a new warped frequency transform that provides enhanced mode extraction. The time-frequency analysis method can obtain the frequency component distribution of signals at different propagation times, and the calculated results can also reflect the general trend of the group velocity dispersion curve, which is helpful for mode identification [19,20,21]. However, it is difficult to apply the time-frequency analysis to the frequency segment where multiple modes cross. Therefore, it is necessary to decouple the aliasing ultrasonic guided waves in the time domain and extract the effective modes for detection [22]. The two-dimensional Fourier transform (2D-FFT) is regarded as a traditional signal-processing and analysis method, which is widely used in various fields [23]. Draudviliene et al. [24] applied 2D-FFT to detect the peak of the spectral amplitude of a specific frequency and proved the effectiveness of this method in Lamb wave spectrum peak detection in aluminium plates through mathematics and experiments. Alleyne and Cawley [25] proposed to use 2D-FFT to obtain the dispersion information from the propagating multi-mode Lamb waves in the frequency-wavenumber (f-k) domain, overcoming the problem of multi-mode and dispersion. Michaels et al. [26] applied the f-k domain analysis method to the research of acoustic field image enhancement damage recognition, effectively decoupled the time-domain waveform, and achieved the visual enhancement effect of defect scattered signal by removing the source wave signal. Gao et al. [27] used 2D-FFT filtering and inverse transformation to extract a single mode, and successfully separated the mode aliasing caused by multiple modes. It can be seen that in the f-k domain, since the propagation characteristics of the wave are related to the frequency component and speed of the wave, 2D-FFT is carried out on the acquired time-domain signals at different propagation distances to obtain mutually separated modes in the f-k domain [28]. However, in practical application, a single mode wave containing only a certain mode is often needed [29], so mode-separation technology emerges. Studies have shown that the ridge extraction method in time-frequency analysis can be used in the process of mode separation and achieve good results [30]. Ridge extraction techniques have also been developed [31,32]. Carmona et al. [33] extracted the time-frequency ridge by applying the penalty function, which is suitable for detecting the time-frequency matrix modal ridge of a single-component signal containing noise. Marzani and De Marchi [34] derived the TFR ridges corresponding to the dispersion curves during the extraction of the experimental data, and extracted the modal group delays by redistributing the spectrograms. An algorithm based on the modal maximum of the time-frequency matrix to extract the time-frequency ridge was proposed by [35]. This algorithm is rapid and simple and is also suitable for the ridge extraction of other two-dimensional matrices. Hu et al. [36] proposed a time-frequency ridge extraction algorithm based on the dynamic programming method, which overcomes the noise interference and local optimization existing in the ridge extraction algorithm. The above algorithm is suitable for ridge extraction of a single-component signal, but in order to apply the ridge extraction algorithm to modal extraction, it is necessary to find a method suitable for multi-component ridge extraction. At the same time, the time-frequency domain of multi-mode signals often contains overlapping parts, and multiple ridges cross each other, causing difficulties in ridge extraction [37]. In the f-k domain, multi-order modes are separated from each other to avoid the appearance of cross parts. Therefore, multi-mode signals can be separated by ridge extraction in the f-k domain, and the time domain signal of a single mode can then be reconstructed from the f-k ridge.

Two-dimensional FFT is often used as a signal processing method for Lamb wave modal analysis in plate structures, but it is rarely reported to be used in rod structures. This paper focuses on the modal excitation and separation of ultrasonic guided waves in the process of propagation by taking the typical cylindrical rod structure as the research object. The finite element software ABAQUS 2016 is used to simulate the propagation process of the ultrasonic guided wave. The finite element model parameters and the form of excitation signal are determined by analyzing the propagation characteristics of ultrasonic guided waves in the structure, and the time-domain signal containing the modal order in a specific range is obtained. We used 2D-FFT to convert the time–space coupling waveform in the rod structure to the f-k domain to achieve waveform decoupling. The specific research idea of mode separation is as follows. Firstly, 2D-FFT is carried out on the two-dimensional amplitude matrix composed of acceleration time-history curves obtained from signal-receiving points at the same distance on the surface of the cylindrical rod, so as to obtain signals in the f-k domain. The separated modes can be seen in the f-k three-dimensional diagram. Then the ridge of each order mode in the three-dimensional diagram is extracted by dynamic programming method, and the amplitude points near each ridge are extracted, which are the separated f-k domain modes. Finally, the 2D-FFT is used to transform each order f-k domain signal back to the time domain signal. The results show that the extracted f-k ridge is consistent with the theoretical dispersion curve. Time-frequency transform is performed on the time-domain signal of a single mode, and the actual arrival time of the mode at a certain frequency is obtained by time-frequency analysis technology, to calculate the group velocity of the guided wave. After calculation, the relative error between the group velocity calculation result and the theoretical group velocity is very small. Therefore, the method of modal excitation and separation adopted in this paper can effectively achieve the excitation and separation of guided wave modes, which provides a guarantee for the subsequent use of a single mode. 

## 2. Theoretical Background and Methodology

### 2.1. Ultrasonic Guided Wave Propagation in Rod Structures

When an ultrasonic wave propagates within a bounded medium, waveform conversion occurs due to the presence of boundaries, resulting in the coupling of waves with different wave speeds and the formation of ultrasonic guided waves [38]. Guided waves exhibit the characteristic of multi-mode propagation, wherein multiple modes exist at the same frequency, each propagating within the medium at different speeds [39]. Guided waves propagated axially can be divided into longitudinal mode L (0, *n*), with only axial and radial direction; torsional mode T (0, *m*), with only circumferential displacement; and bending mode F (*n*, *m*), with displacement in all three directions of axial, radial and circumferential direction according to the difference between vibration direction and propagation direction. *n* is the circumferential order of the mode, and *m* is the modulus. 

**Figure 1:**
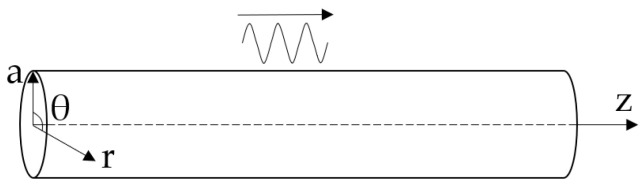
Infinitely long cylindrical rod of radius *a*.

Set the radius of an infinitely long cylinder with a free surface as *a*, and place it along the axis *z*. Take the cylindrical coordinate system, as shown in Figure 1.

In the longitudinal mode, only radial and axial displacement components exist. The circumferential component of displacement ${u=(u}_{r}{,\;0,\;u}_{z})$ is zero, and the displacements of all points on the rod are symmetric about the central axis of the rod. By introducing the boundary condition $\sigma_{rr}=\sigma_{rz}=0$ (at *r* = *a*), the Pochhammer frequency equation can be obtained by solving the wave equation:

(1) $$\frac{2\alpha }{a}(\beta^{2}{+k}^{2})J_{1}\left(\alpha a\right)J_{1}(\beta a)-{\left(\beta^{2}-k^{2}\right)}^{2}J_{0}(\alpha a)J_{1}(\beta a)-4k^{2}\alpha \beta J_{1}(\alpha a)J_{0}(\beta a)=0$$

where $\alpha^{2}=\frac{\omega^{2}}{c_{L}^{2}}\;-\;k^{2}$, $\beta^{2}=\frac{\omega^{2}}{c_{T}^{2}}\;-\;k^{2}$, ω is the circular frequency, *k* is the wavenumber, *J*_0_ and *J*_1_ are the first- and second-order of Bessel function, $c_{L}$ is the dilatational wave velocity, and $c_{T}$ is the shear wave velocity.

**Figure 2:**
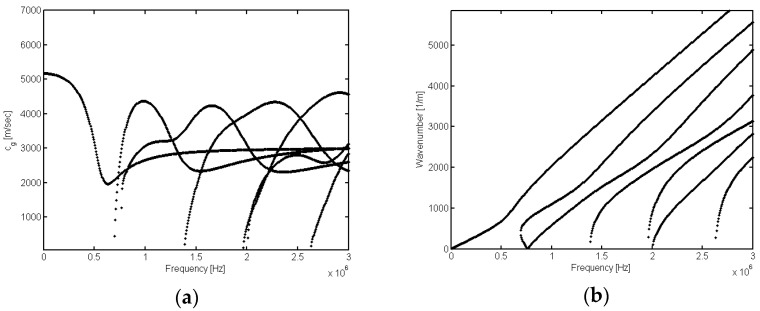
Ultrasonic guided wave dispersion curve of the cylindrical rod: (**a**) group velocity curve; (**b**) f-k curve.

By numerically solving the transcendental equations of *ω* and *k*, it becomes evident that a single circular frequency *ω* can correspond to multiple wavenumbers *k*. The frequency equation of ultrasonic wave propagating in a rod waveguide has the characteristics of multimode and dispersion. Figure 2 shows the dispersion curve of longitudinal modes of ultrasonic guided waves in a cylindrical rod with a radius of 2.6 mm. In Figure 2a, it can be observed that at a specific frequency, multiple longitudinal guided wave modes exist within the rod, each propagating at a distinct group velocity. As the frequency increases, the number of modes and the complexity of the signal also increase. Figure 2b illustrates the dispersion curve in the f-k domain; it is observed that modes of each order separate from each other without the interference of cross terms, which provides a theoretical basis for the subsequent mode separation.

### 2.2. Basic Theory of 2D-FFT

Due to the multi-mode property of the ultrasonic guided wave, the time domain signal of each signal-receiving point is a complex wave coupled by multiple modes. Furthermore, the ultrasonic guided wave displays dispersion, where the propagation velocity of each mode varies with frequency. Consequently, extracting meaningful information solely from the time domain signals is challenging. In this regard, 2D-FFT extends one-dimensional Fourier transform directly to multidimensional signals, making it an effective method for analysing signals in the time–space domain. By 2D-FFT, the waves coupled together in the time domain can be separated from each other in the f-k domain, allowing for the waveform decoupling. Then the desired modes can be separated in the f-k diagram, enabling the study of the dispersion phenomenon exhibited by each mode of the ultrasonic guided wave:

(2) $$H(f,\;k)=\int_{-\infty }^{+\infty }\int_{-\infty }^{+\infty }h(t,\;x)e^{-i(kx+\omega t)}dxdt$$

where h(t, x) is the time-domain signal of each equidistant point on the surface of the member, *f* is the frequency and the wavenumber $k=\frac{2\pi }{\lambda }$, and λ is the wavelength. In the actual calculation, the discrete 2D-FFT is utilized. Multiple equidistant signal reception points are established at the receiving end, and time-domain signals are simultaneously recorded at these points, forming a time–space matrix h(t, x). The fundamental principle of 2D-FFT is to perform two one-dimensional Fourier transforms. The implementation process begins with conducting a one-dimensional Fourier transform on the time-domain signal at each equidistant signal reception point, and obtaining the frequency spectrum of each location to form the frequency–space matrix H(f, k). Then the frequency is fixed, and the space vector at each frequency is transformed by one-dimensional Fourier transform to form the f-k matrix H(f, k). The discrete calculation of the 2D-FFT is as follows:

(3) $$H(f,\;k)=\overset{N_{x}}{\underset{n_{x}=1}{\sum }}\overset{N_{t}}{\underset{n_{t}=1}{\sum }}h(t,\;x)e^{-i(kn_{x}+\omega n_{t})}$$

where *N_x_* and *N_t_* are the sequence length of discrete space and discrete time, *n_x_* is the discrete space sequence, and *n_t_* is the discrete time sequence. By projecting the resulting f-k matrix onto the f-k plane, an f-k graph can be obtained. 

### 2.3. Dynamic Programming Method

Ridge extraction technology based on dynamic programming is introduced into the f-k domain to overcome the challenges caused by the crossing of multiple modes in the time-frequency domain. This approach combines the strengths of the modal maximum method and the penalty function ridge extraction method. The process begins by initially extracting the ridge based on the modal maximum value. Then, the penalty function proposed by Carmona et al. [31] is employed to smooth out any discontinuities or abrupt changes in the ridge, ensuring a more consistent and accurate representation. By applying the penalty function, the ridge-extraction problem is transformed into an optimization problem, so as to reduce the impact of simultaneous mode presence and close space.

The specific steps of ridge extraction for discrete signals are as follows:

1. In the first step, the initial ridge is extracted based on the modal maximum method, which involves identifying the maximum values corresponding to the desired mode in the signal. This initial ridge provides a starting point for further refinement.
  (4) $$K(f)=\max[{\left|H(f,\;k)\right|}^{2}]$$
2. The second step involves optimizing the ridge line using the penalty function, allowing for the determination of the optimal ridge line.
  (5) $$\hat{K}(f_{tp})=\overset{N}{\underset{f_{tp}=2}{\sum }}[-{\left|H(f_{tp},k)\right|}^{2}+\lambda_{n}{|K(f_{tp})-K(f_{tp}-1)|}^{2}]$$
  where K(f) is the initial ridge, $\hat{K}$ is the optimal wave number curve, *N* is the signal length, $f_{tp}$ is the frequency translation parameter of the discrete signal, and $\lambda_{n}$ is the normal number of smoothness of the equilibrium curve.

Assume that the optimal subpath of the first *i* steps has been determined, the terminus passes through point (K(*i*), *i*) (where K(*i*) = *p*), and its corresponding penalty function is $\hat{K}\left(p,\;i\right)$; the next optimal path passes through point (K(*i* + 1), *i* + 1) (where K(*i* + 1) = *q*), then the corresponding penalty function is:

(6) $$\hat{K}\left(q,\;i+1\right)=\min[\hat{K}{\left(p,\;i)\;-\;|K(q,\;i+1)\right|}^{2}+\lambda_{n}{\left|q\;-\;p\right|}^{2}]$$

To determine the point corresponding to the optimal path *i* + 1 step, all candidate ridge points at this point need to be compared with the *i* + 1 step path composed of the optimal subpath of the first *i* steps. The point with the minimum penalty function value is retained, so as to obtain the optimal subpath of the first *i* + 1 step. By iteratively evaluating and updating the penalty function values, the algorithm seeks to find the most favourable path through the ridge points, ensuring an accurate and consistent extraction of the ridge.

When the f-k ridge exhibits a stable variation trend, it suggests a small gradient of wave number with respect to frequency and consequently a low penalty function value. Conversely, when there is a high local energy peak in the f-k domain caused by noise, the wave number experiences significant changes, resulting in an increase in the penalty function value. Therefore, the optimal ridge corresponds to the minimum value of the penalty function across the entire f-k domain, representing the desired modal peak curve to be extracted. For the multi-ridge extraction problem, the previous peak ridge can be set to zero, and the above steps can be repeated accordingly. This iterative process helps identify and isolate individual ridges effectively.

## 3. Finite Element Model of the Cylindrical Rod

The finite element model of a cylindrical rod with length *L* of 520 mm and radius *r* of 3 mm is established in ABAQUS. The material parameters of the finite element model are shown in Table 1.

### 3.1. Description of the Simulation

In order to obtain multi-modal data for one-time excitation, a wide-band triangular wave is used as the broadband excitation signal. The triangular pulse force *F*(*t*) is expressed by the following formula:

(7) $$\;F(t)=\left\{\begin{array}{l} a_{f}t,\;0\;\leq \;t\;\leq {\;t}_{0}\\ \frac{1}{2}{a_{f}(3t}_{0}{\;-\;t),\;t}_{0\;}\leq \;t\;\leq {\;3t}_{0}\\ {0,\;t\;>\;3t}_{0} \end{array}\right.$$

where *t*_0_ is the time corresponding to the maximum applied load, and *a_f_t* is the maximum applied force.

**Figure 3:**
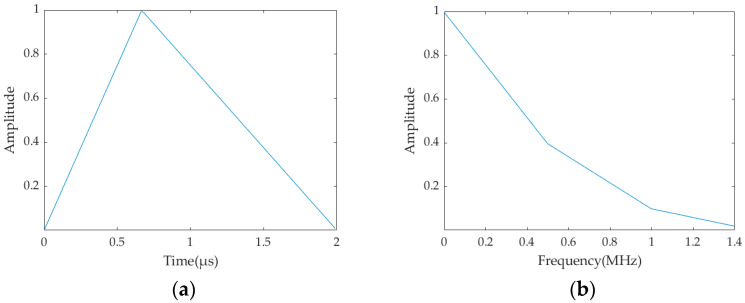
Triangular wave pulse excitation: (**a**) time domain; (**b**) frequency domain.

In this study, the excitation and separation of the first four longitudinal modes are conducted. The excitation signal is set to have a frequency range of 0~1.4 MHz, and *t*_0_ in the triangular wave is set as $\frac{2}{3}\;\mu s$. In order to facilitate signal recording by finite element software, both the sampling frequency of the excitation signal and the recording frequency of the signal receiving points are set as $\frac{1}{{1.7\;\times \;10}^{-8}}\;\mathrm{Hz}$. The time domain and spectrum of the triangular wave are shown in Figure 3.

The selection of cell mesh size is a critical step in the process of finite element simulation. The size of the mesh depends on factors such as the structure types, material characteristics, and the specific research objectives, and it directly affects the quality of the mesh produced. Using a mesh size that is too large can introduce significant errors, while using a mesh size that is too small can result in slow computation. It is proved by the theory that the wave characteristics of guided waves can be simulated more accurately when the mesh size of the element is no smaller than 1/7 of the shortest wavelength in the excited mode, leading to more precise simulation results. Therefore, the maximum mesh size follows the principle of no more than $\frac{\lambda_{\min}}{7}$, where $\lambda_{\min}$ is the minimum wavelength of the excited mode.

**Figure 4:**
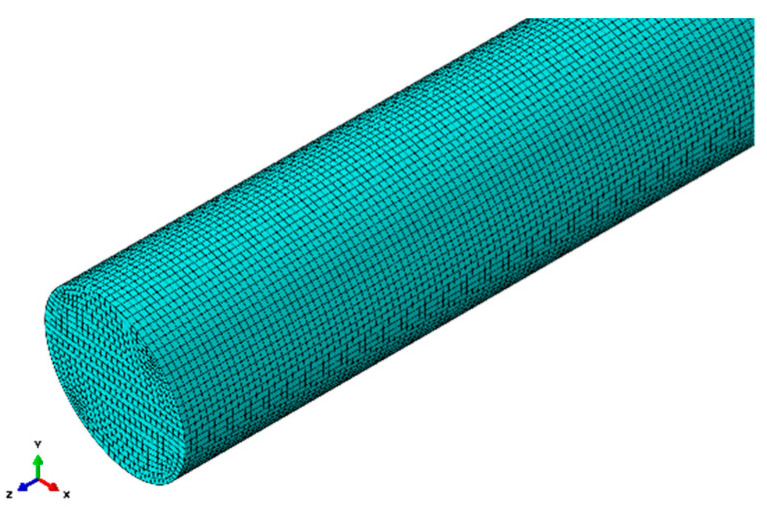
Cylindrical rod finite element meshing diagram.

In this paper, the excitation signal is centred at a frequency of 0.3 MHz. At this frequency, the minimum wavelength corresponding to the lowest group velocity is $\lambda_{\min}=\frac{v_{g}}{f_{c}}=7$ mm, where *v_g_* represents the minimum group velocity at a given frequency, and *f_c_* is the centre frequency of interest. Based on this, the theoretical maximum mesh size is selected as 1 mm. Figure 4 illustrates the schematic diagram of the mesh division in the finite element model.

In dynamic transient analysis, the accuracy of the numerical solution for the wave propagation process relies on the integral step size. A smaller step size provides a higher calculation precision and easier convergence, but it also requires more computational resources. The structural response can be considered as a combination of various modes, and the minimum integration step should be capable of resolving the highest mode in the combination of structural response modes. 

### 3.2. Finite Element Model

The discrete 2D-FFT is used in the actual calculation, requiring adherence to the Nyquist sampling theorem in both the time and space domains to avoid signal aliasing. In the spatial domain, the spacing between samples should be no greater than half of the shortest wavelength. However, in practical scenarios, the sampling frequency needs to be approximately five times higher to ensure accurate signal recording. According to Section 3.1, the excitation frequency necessary to stimulate the desired higher-order mode is 0.3 MHz. Consequently, the maximum sampling interval should not exceed the corresponding spatial wavelength, which amounts to Δd=λmin5=1.2 mm. In this study, the response signals are collected at the nodes, and the grid size is set to 1 mm, satisfying the requirements of the sampling theorem. Therefore, the distance between two adjacent signal points, as set in this paper, is 1 mm. 

**Figure 5:**
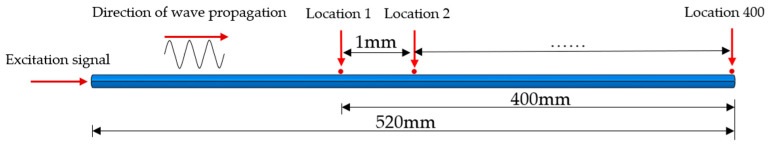
Load location and signal reception locations.

As mentioned previously, the axial symmetric displacement field structures, such as straight rod structures, exhibit prominent longitudinal modal characteristics. To selectively excite the longitudinal guided wave mode, a concentrated load is applied at the centre of the circular free end face along the axial direction of the rod. Signal-receiving points are positioned along the axial surface of the rod, with a spacing of 1 mm and a total of 400 nodes, at a distance of 120 mm. These nodes are utilized to capture the transient acceleration signals. The loading location and signal-receiving locations are shown in Figure 5.

**Figure 6:**
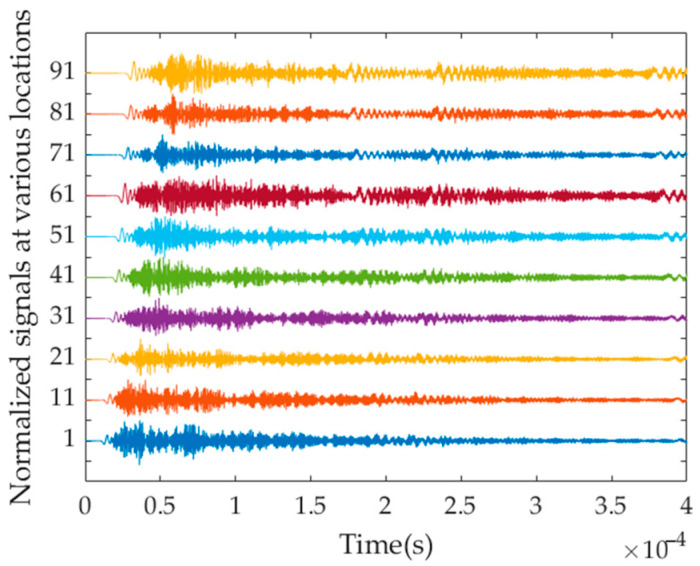
Normalized acceleration time domain signals at various locations.

The acquisition frequency *f_s_* of the acceleration signal is selected as 100 MHz, with an acquisition duration *t* of 0.4 ms, resulting in a total of 40,000 sampling points. Figure 6 shows the amplitude diagram of normalized transient acceleration signals from ten signal-receiving points, where the vertical axis represents the signal-receiving location. It can be observed from the figure that due to the superposition of multiple modes, it is impossible to identify individual modes based on the time domain signals alone.

## 4. Analysis in the f-k Domain

### 4.1. Cylindrical Rod Ridge Extraction

**Figure 7:**
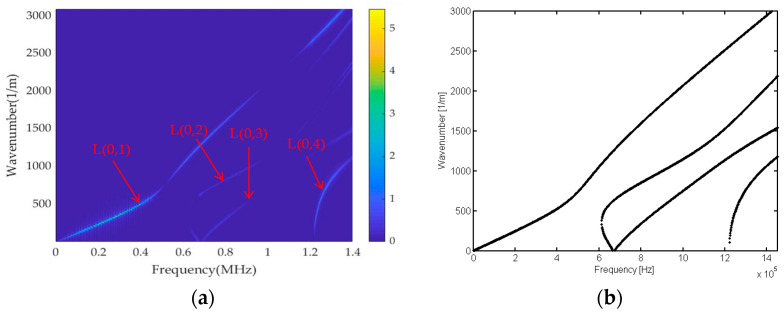
Comparison of cylindrical rod dispersion curves: (**a**) 2D-FFT f-k spectrum; (**b**) theoretical f-k spectrum.

The transient acceleration signals obtained from the finite element model are subjected to 2D-FFT, and the results are presented in Figure 7a, displaying four distinct energy lines. Figure 7b depicts the theoretical dispersion curve, demonstrating a high degree of agreement between the two. This suggests that the f-k distribution diagram can effectively capture the dispersion characteristics of the cylindrical rod structure, providing an accurate description of its behaviour.

**Figure 8:**
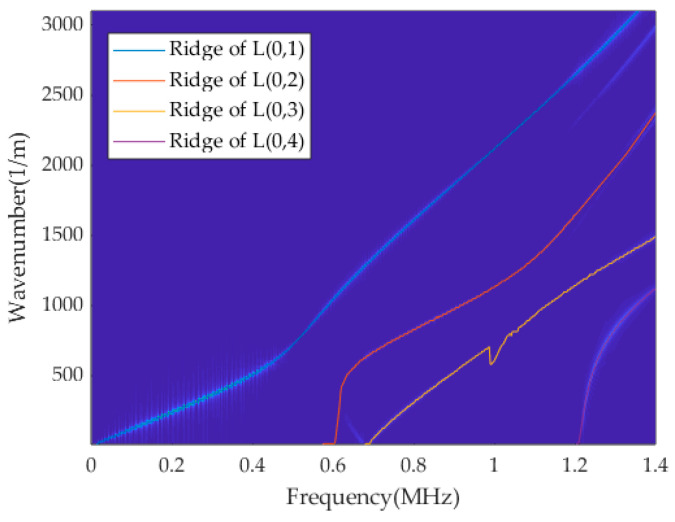
Ridges of the multi-mode signal containing the L(0, 1), L(0, 2), L(0, 3) and L(0, 4) modes.

The f-k domain graph in Figure 7 undergoes ridge extraction using dynamic programming, and the corresponding results are presented in Figure 8. As expected, the energy ridges corresponding to the fourth mode are distinctly identified, confirming that the fourth mode can be successfully separated by the presence of four ridges.

**Figure 9:**
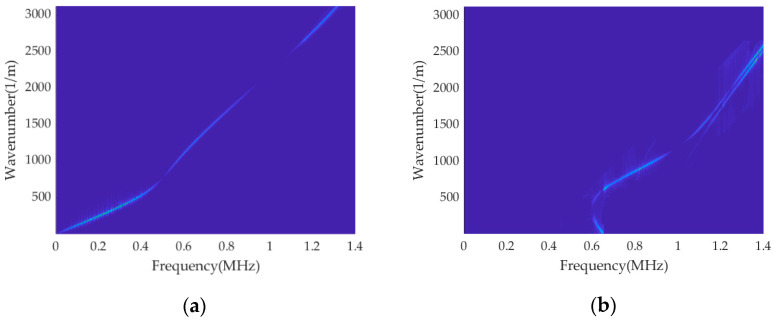

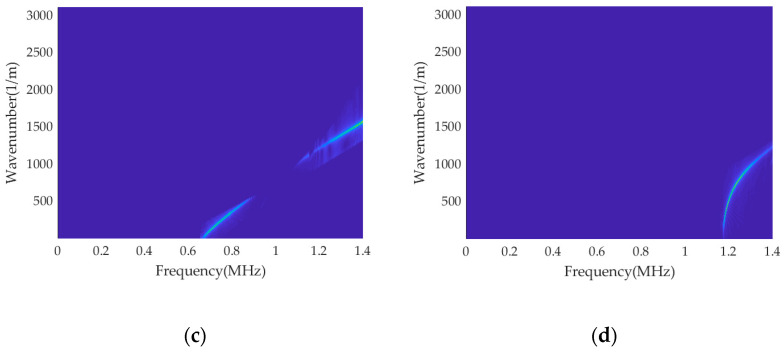
Multi-mode separation results: (**a**) f-k spectrum of Mode L(0, 1); (**b**) f-k spectrum of Mode L(0, 2); (**c**) f-k spectrum of Mode L(0, 3); (**d**) f-k spectrum of Mode L(0, 4).

According to the ridge-extraction results in Figure 8, the mode separation in the multi-mode f-k domain is conducted, and the single mode after separation is obtained, as shown in Figure 9. It is evident that the fourth mode has been successfully separated. Furthermore, the separated single mode is unaffected by other modes, resulting in enhanced brightness in the f-k domain and achieving a visually improved representation.

**Figure 10:**
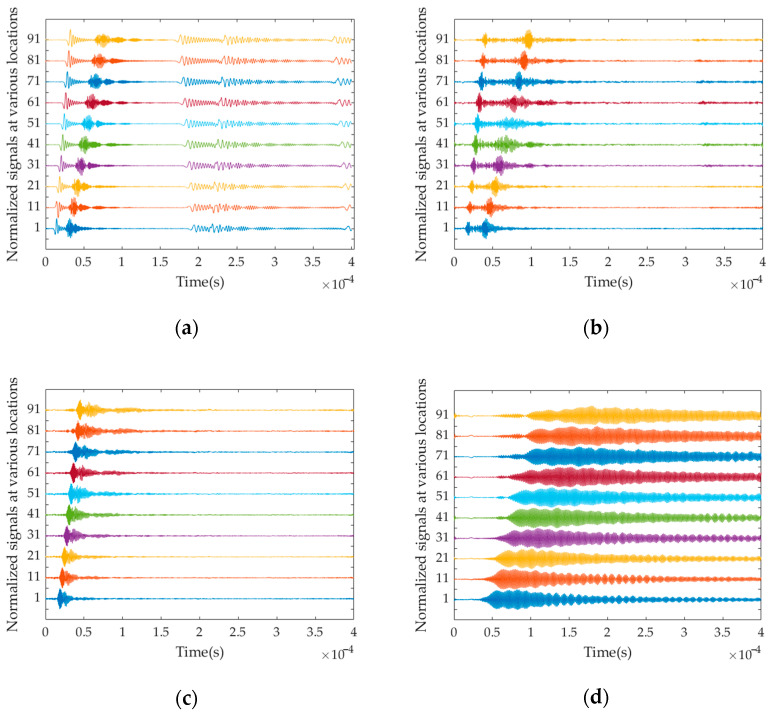
Single-mode normalized acceleration time-domain signals at various locations: (**a**) time-domain signal of Mode L(0, 1); (**b**) time-domain signal of Mode L(0, 2); (**c**) time-domain signal of Mode L(0, 3); (**d**) time-domain signal of Mode L(0, 4).

The time domain signal of a single mode can be obtained by performing a two-dimensional inverse Fourier transform on the single mode extracted from the multi-mode f-k ridge, as shown in Figure 10. It is evident that four distinct sets of regular time-domain signals can be obtained after the f-k separation of the original aliased time-domain signals that contain four modes.

As can be seen from Figure 10, all modes are successfully separated in the time domain. To validate the correctness of the calculation, the middle mode L(0, 2) is selected for verification. It is well-known that the guided wave in the waveguide propagates in the form of the wave packet, and the propagation velocity of each wave packet corresponds to the group velocity of the guided wave. In the case of multiple modes, the wave packets intersect and overlap with each other, making it difficult to determine the group velocity solely based on the position of the wave packet in the time domain. By employing mode-separation techniques, the aliased time-domain signal can be divided into individual mode signals, and each only contains single-mode information. This allows researchers to extract the propagation signal of the wave packet associated with each mode from the time-domain signal. However, it should be noted that the group velocity of each mode varies with time due to the discussed dispersion property. To obtain the time variation of each guided wave mode, it is necessary to perform the time-frequency transform on the individual mode time-domain signal. In this study, the S-transform is employed to analyse the reconstructed time-domain signal of L(0, 2).

**Figure 11:**
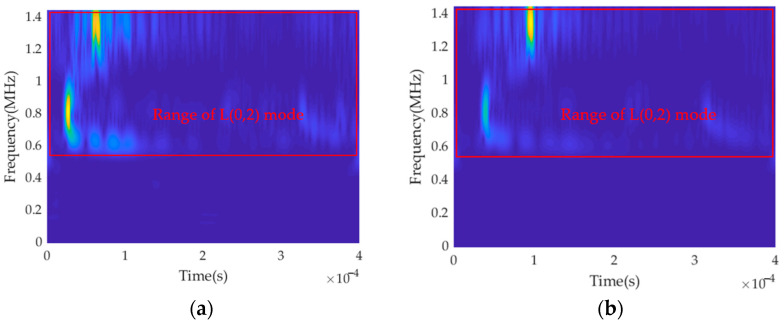
S-transform of L(0, 2) mode at different locations: (**a**) Location ‘50’; (**b**) Location ‘101’.

Figure 11 shows the S-transform of time-domain signals at Location ‘50’ and Location ‘101’. The obtained distribution range of the L(0, 2) mode aligns with the theoretical range, spanning 0.6 MHz to 1.4 MHz. This confirms the accuracy and validity of the mode-separation technique, as it successfully separates the desired mode and reveals its frequency range.

**Figure 12:**
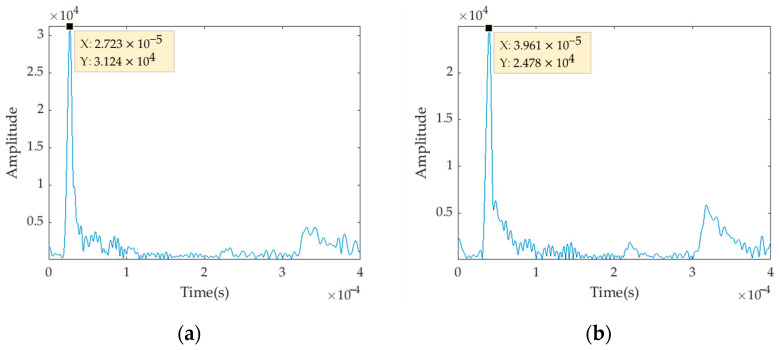
Time-domain diagram at frequency of 0.8375 MHz: (**a**) Location ‘50’; (**b**) Location ‘101’.

To validate the accuracy of the mode-separation results, the group velocity at a frequency of 0.8375 MHz is compared with the theoretical group velocity. The group velocity can be calculated using the formula $v_{g}=\frac{\Delta d}{\Delta t}$, where Δ*t* is the time difference corresponding to the maximum amplitude of the signals at that frequency. The corresponding time values at Location ‘50’ and Location ‘101’ for the frequency of 0.8375 MHz are indicated as $t_{1}$ and $t_{2}$ in Figure 12.

**Figure 13:**
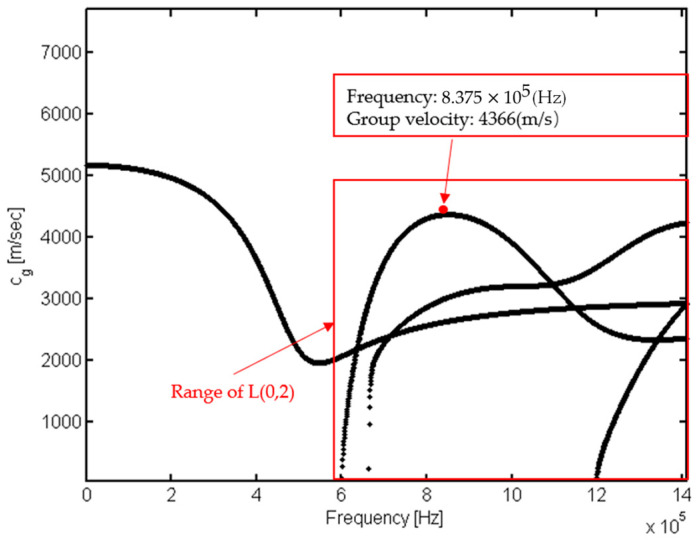
Theoretical group velocity value at frequency of 0.8375 MHz.

The group velocity calculated according to the above results is: $v_{g}=\frac{\Delta d}{\Delta t}=4038.7\;m/s$. Figure 13 shows the theoretical group velocity value at this frequency. The error between the numerical results and the theoretical results is: $\delta =\frac{\left|v_{g\_\mathrm{theory}}\;-\;v_{g\_\mathrm{computation}}\right|}{v_{g\_\mathrm{theory}}}=7.49\%.$ The error is small, which proves the reliability of the separation results.

### 4.2. Noise Effect

In the practical application of ultrasonic guided wave monitoring of the circular rod, because the amplitude of the guided wave signal is quite small, it is easy to be submerged by noise. If the ultrasonic guided wave signal is directly analysed by spectrum analysis, then there will be a certain difference in the amplitude of the actual signal. At the same time, in the actual acquisition and transmission of the signal, due to the interference of the external environment and the influence of instruments, there will inevitably be noise mixed into it, and noise is an important factor affecting the detection and identification performance of the target signal. Especially when analysing some high-precision data, even very weak noise will have a great impact on the analysis results. Therefore, it is necessary to discuss whether the signal with noise will affect the ridge extraction results based on the dynamic programming method.

**Figure 14:**
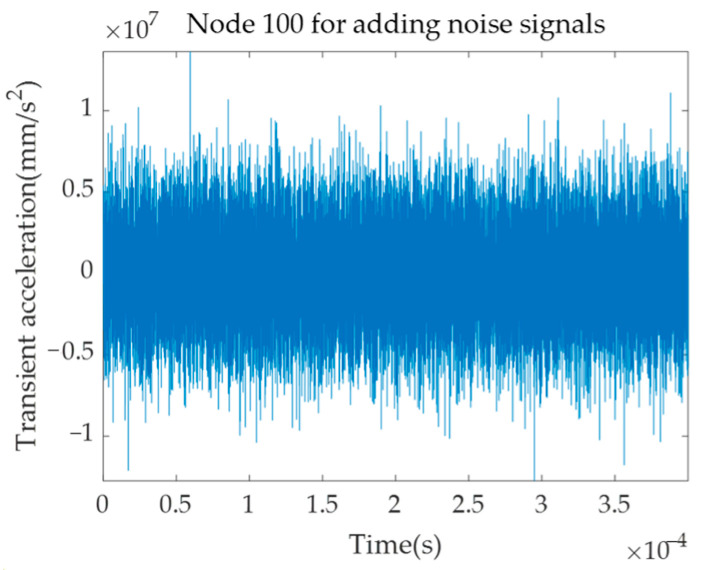
Time-domain diagram of Location ‘100’ with additional noise.

**Figure 15:**
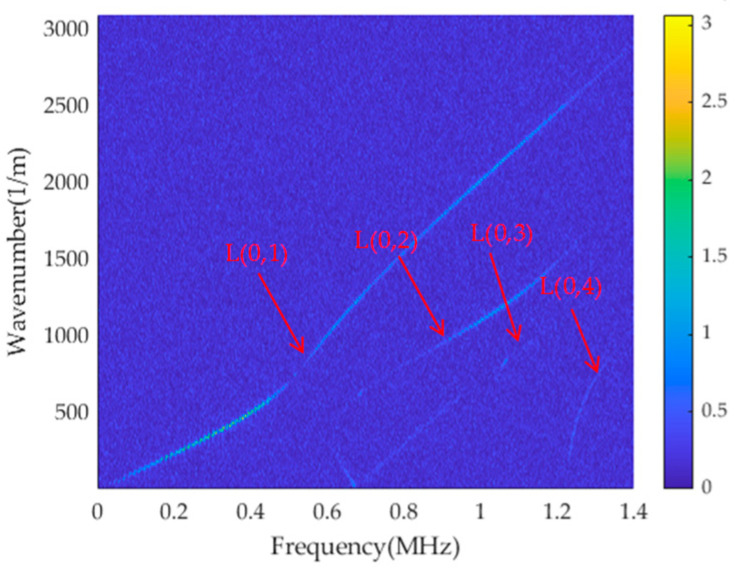
Cylindrical rod dispersion curves by 2D-FFT f-k spectrum with noise.

**Figure 16:**
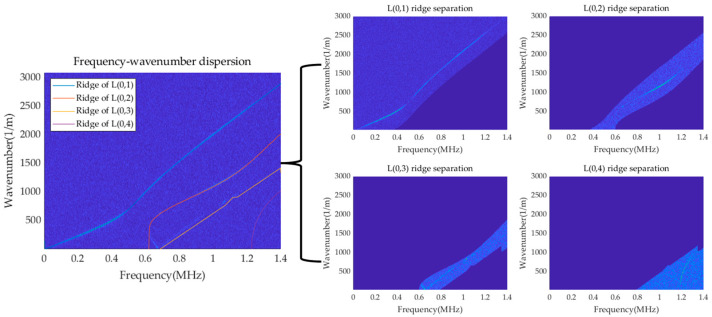
Results of ridge extraction of the cylindrical rod with noise.

A triangular wave excitation signal is applied at one end of the rod, and the finite element simulation parameters and signal receiving position are set as described above. A Gaussian white noise with a signal-to-noise ratio of −25 is added to the time–space matrix composed of the obtained transient acceleration signals, and the time-domain diagram of Location ‘100’ is shown in Figure 14. The 2D-FFT is performed on the signal after noise processing, and the results obtained are shown in Figure 15. According to the dynamic programming method, ridge extraction is carried out for the f-k domain figure, and mode separation is performed on the multi-mode f-k domain. The separated results are shown in Figure 16.

It can be seen from the above figure that due to the influence of additional noise, the results of the energy ridges shown on the f-k domain diagram become worse; as the number of mode orders increases, the higher-order ridge is not shown clearly. Based on the dynamic programming method of circular rod under the action of ultrasonic guided waves, the noise has little effect on the ridge-extraction results, and the energy ridges of the four modes can be well identified and separated, and the separated single mode is not affected by other modes. Although the energy of the higher-order modes in the f-k domain diagram after 2D-FFT is smaller and less clear than that of the fundamental mode due to the influence of noise, the method utilized in this paper can still identify the energy ridge of the higher-order modes well, and even if there is a certain deviation, the effect on the overall results can be ignored.

## 5. Conclusions

This paper investigates the propagation characteristics of ultrasonic guided waves excited by a broadband signal in a rod structure. Furthermore, the application of 2D-FFT is employed to explore the wave propagation within the rod structure. To address the challenge of separating multi-mode signals in the time domain, a proposed method converts the coupled ultrasonic guided waves from the time domain to the f-k domain, enabling their effective decoupling. Firstly, the propagation of the ultrasonic longitudinal guided wave is simulated using ABAQUS. According to the axial acceleration responses of each point collected synchronously, a three-dimensional amplitude matrix with time–space information is obtained. Through the 2D-FFT of the amplitude matrix, the signals in the time–space domain are transformed into the f-k domain, providing a clear f-k diagram. Then the ridge in the f-k domain is extracted using the dynamic programming method. The range of each mode is determined based on the location of the ridge, and the two-dimensional inverse Fourier transform is performed to obtain the time-domain signal corresponding to a single mode. Finally, the influence of noise on the ridge-extraction results based on the dynamic programming method is discussed. This paper thoroughly investigates the excitation and separation of multi-order longitudinal guided wave modes, building upon an in-depth study of the propagation characteristics of ultrasonic guided waves in the cylindrical rod. Successful excitation and separation of higher-order modes lay a solid foundation for future applications of single modes.

## Figures and Tables

**Table 1:** Material parameters of finite element model.

Material	Density (kg/m^3^)	Elastic Modulus (GPa)	Poisson Ratio
Steel	7850	210	0.29

## Data Availability

Not applicable.
